# Pyrolized Diatomaceous Biomass Doped with Epitaxially Growing Hybrid Ag/TiO_2_ Nanoparticles: Synthesis, Characterisation and Antibacterial Application

**DOI:** 10.3390/ma16124345

**Published:** 2023-06-13

**Authors:** Weronika Brzozowska, Izabela Wojtczak, Viorica Railean, Zhanar Bekissanova, Grzegorz Trykowski, Bogusław Buszewski, Myroslav Sprynskyy

**Affiliations:** 1 Institute of Marine and Environmental Sciences, Doctoral School, University of Szczecin, Mickiewicza 16, 70-383 Szczecin, Poland; weronika.brzozowska@phd.usz.edu.pl; 2 Department of Environmental Chemistry and Bioanalytics, Faculty of Chemistry, Nicolaus Copernicus University in Torun, 7 Gagarina Str., 87-100 Torun, Poland; izabelawojtczak1991@gmail.com (I.W.); bbusz@umk.pl (B.B.); 3 Department of Infectious, Invasive Diseases and Veterinary Administration, Institute of Veterinary Medicine, Nicolaus Copernicus University in Torun, Gagarina 7, 87-100 Torun, Poland; viorica.railean@umk.pl; 4 Interdisciplinary Center for Modern Technologies, Nicolaus Copernicus University in Torun, Wilenska 4, 87-100 Torun, Poland; 5 Faculty of Chemistry and Chemical Technology, Al-Farabi Kazakh National University, 050040 Almaty, Kazakhstan; bekisanova@gmail.com; 6 Center of Physical-Chemical Methods of Research and Analysis, 050012 Almaty, Kazakhstan; 7 Faculty of Chemistry, Nicolaus Copernicus University in Torun, Gagarina 7, 87-100 Torun, Poland; tryki@umk.pl

**Keywords:** diatom biomass, biosynthesis, epitaxial growths, epitaxial Ag/TiO_2_ nanoparticles, hybrid nanostructures, antibacterial activity, photoluminescence properties

## Abstract

In the pursuit of innovative solutions for modern technologies, particularly in the design and production of new micro/nanostructured materials, microorganisms acting as “natural microtechnologists” can serve as a valuable source of inspiration. This research focuses on harnessing the capabilities of unicellular algae (diatoms) to synthesize hybrid composites composed of AgNPs/TiO_2_NPs/pyrolyzed diatomaceous biomass (AgNPs/TiO_2_NPs/DBP). The composites were consistently fabricated through metabolic (biosynthesis) doping of diatom cells with titanium, pyrolysis of the doped diatomaceous biomass, and chemical doping of the pyrolyzed biomass with silver. To characterize the synthesized composites, their elemental and mineral composition, structure, morphology, and photoluminescent properties were analysed using techniques such as X-ray diffraction, scanning and transmission electron microscopy, and fluorescence spectroscopy. The study revealed the epitaxial growth of Ag/TiO_2_ nanoparticles on the surface of pyrolyzed diatom cells. The antimicrobial potential of the synthesized composites was evaluated using the minimum inhibitory concentration (MIC) method against prevalent drug-resistant microorganisms, including *Staphylococcus aureus*, *Klebsiella pneumonia,* and *Escherichia coli*, both from laboratory cultures and clinical isolates.

## 1. Introduction

Numerous epidemiological analyses have consistently demonstrated a strong association between microbial contamination and an elevated risk of transmitting infectious diseases. Various pathogens, including bacteria, fungi, viruses, and protozoa, can give rise to a range of diseases. The effectiveness of antibiotics against pathogenic microorganisms has been well-established and has played a pivotal role in saving numerous lives over the past century [1,2,3]. Regrettably, the frequent and excessive utilization of conventional antibiotics has led to widespread multidrug resistance in bacterial infections [4,5,6]. Consequently, there is an urgent need to develop antibacterial agents that are both effective and safe [7,8,9]. To address this challenge, researchers have turned their attention to nanocomposites, which represent a cost-efficient and efficient material for treating various diseases [10,11,12]. Notably, silver nanoparticles have received considerable attention due to their potent antimicrobial properties and extensive use in medicine, owing to their strong antimicrobial activity and relatively favourable biocompatibility [13,14,15]. However, the cytotoxic and bactericidal effects of AgNPs present a significant conundrum, as the mechanisms responsible for the toxicity of nanoparticles towards microorganisms may also affect mammalian cells. Human cell toxicity and the limited efficiency of AgNP penetration through bacterial biofilms are the primary obstacles hindering their biomedical applications [16]. Therefore, the pursuit of novel nanocomposites containing nanoscale silver represents a considerable challenge for contemporary science, as it necessitates the minimization of AgNPs’ toxic effects, a crucial prerequisite for their medical utilization. For this reason, it would be necessary either to exclude silver nanoparticles from antibacterial materials or to minimize their toxic effect, which is a key prerequisite for the medical use of AgNPs. Considering the first solution, selenium-containing materials could be a very good alternative to silver nanoparticles due to their bioactivity, biocompatibility, relatively low toxicity, and the low price of the Se element [17]. Regarding the second option, hybrid composites incorporating semiconductor nanoparticles and reduced silver appear to offer an ideal solution to this predicament [18,19,20]. In the production of such materials, an extensive range of raw substances, including metal oxides such as copper oxide (CuO) [21], zinc oxide (ZnO) [22], and titanium oxide (TiO_2_) [23], have been employed as semiconductor matrices for Ag nanoparticles. Among them, the Ag/TiO_2_ nanocomposite has emerged as a promising antibacterial material with low biotoxicity. Studies have demonstrated the efficacy of nanocomposite materials comprising silver and titanium dioxide against a variety of Gram-positive bacteria (*B. subtilis*, *S. aureus*, MRSA), Gram-negative bacteria (*E. coli*, *K. pneumoniae*, *P. aeruginosa*), and fungi (*C. albicans*) [24,25,26,27]. TiO_2_ nanoparticles are commonly employed in photocatalytic antibacterial applications due to their affordability, chemical stability, and non-toxicity to humans [28]. However, as TiO_2_ is a semiconductor with a wide band gap of 3.26 eV for anatase [29], it can only be activated under UV radiation, which accounts for approximately 5% of solar energy, while visible light encompasses about 45% of solar energy. Nevertheless, studies have demonstrated that Ag nanoparticles enhance the photoactivity of TiO_2_ by reducing the recombination rate of its photo-excited charge carriers. Consequently, silver-doped TiO_2_ significantly enhances the photocatalytic inactivation of bacteria [30,31]. However, Ag/TiO_2_ nanoparticles tend to aggregate, leading to the release of nanoparticles into the environment during the purification process [32]. To circumvent this issue and safely utilize Ag/TiO_2_ as an antibacterial agent, it is crucial to apply it onto a suitable substrate characterized by high biocompatibility and non-toxicity, as well as excellent sorption properties, to prevent the release of the toxic agent into the environment. Unicellular microalgae, specifically diatoms, represent an ideal substrate for such nanocomposites. Since the 1860s, when Alfred Nobel utilized diatomaceous earth to stabilize nitro-glycerine during dynamite production [33], the extraordinary adsorbent potential of diatoms has been recognized. Diatoms possess a natural ability to synthesize amorphous silica (silica exoskeletons) with a hierarchical, three-dimensional structure [34]. These “natural microtechnologists” can serve as a valuable source of inspiration in the design and production of novel inorganic nanocomposites. Silica shells from over 100,000 known diatom species exhibit an incredible array of intricate, three-dimensional silica structures with unique, open-work morphology. These exceptional properties of diatoms have garnered significant interest due to their potential utilization as effective adsorbents or models. Previous studies have reported on the zeolitisation of hierarchical diatom nanostructures [35] and the utilization of their three-dimensional exoskeleton structures as templates in the synthesis of nanomaterials through chemical methods [35,36,37,38].

This study presents the synthesis, characterisation, and antibacterial activity of Ag/TiO_2_/pyrolyzed diatomaceous biomass of *Pseudostaurosira trainorii* (DBP) nanocomposites for the first time. The nanocomposites were characterized using X-ray diffraction (XRD) and photoluminescent properties (PL). Scanning and transmission electron microscopy coupled with energy-dispersive X-ray spectrometry (SEM-EDS and STEM-EDS) were employed to evaluate the morphology, size distribution, and particle size of the nanocomposite. Our synthesized Ag/TiO_2_/DBP nanocomposites exhibited excellent antimicrobial efficacy against *Staphylococcus aureus*, *Klebsiella pneumoniae*, and *Escherichia coli*.

## 2. Materials and Methods

### 2.1. Metabolically Doping Diatomaceous Biomass with Titanium

The pure strain of the diatom species *Pseudostaurosira trainorii* was obtained from the Culture Collection of the Institute of Oceanography of at the University of Gdańsk, specializing in Baltic Sea Algae. A 25-L photobioreactor was utilized, filled with Guillard f/2 medium, and adjusted to a final pH of 8.4. The diatom culture was incubated under a 24 h light regime provided by two 1500 lux fluorescent lamps, maintaining a temperature of 20 °C. The initial concentration of soluble silicon (Na_2_SiO_3_·5H_2_O) in the medium was 7 mg Si/L, while the initial concentration of soluble titanium (TiCl_3_) was 50 mg Ti/L. The growth rate of the diatomaceous biomass in the cultures was assessed daily by measuring the optical density using a Biosan DEN-1B densitometer at a wavelength of 565 nm. Additionally, the growth of diatom cells was monitored using a Light Microscope Axio Observer.D1 (Zeiss, München-Hallbergmoos, Germany) at a magnification ranging from 40× to 100×.

### 2.2. Preparation of AgNPs/TiO_2_/Pyrolized Diatomaceous Biomass Composites

The obtained diatomaceous biomass was initially metabolically doped with titanium and subsequently subjected to pyrolysis. The diatomaceous biomass (DB) was placed in a ceramic crucible, which was then inserted into a quartz tube. The tube was evacuated and filled with argon gas, and the biomass was heated under a continuous flow of argon (Ar 5.0) at a rate of 100 °C/h in a programmable horizontal tube furnace (Czylok, Jastrzębie-Zdrój, Poland). The temperature was raised to 800 °C and maintained for 2 h. This process yielded the TiO_2_/DBP composite.

To synthesize the AgNPs/TiO_2_/DBP composite, the pyrolyzed diatomaceous biomass previously metabolically modified by titanium (TiO_2_/DB) was impregnated with a solution of silver nitrate. A solution of AgNO_3_ (Sigma Aldrich, Burlington, MA, USA) with a concentration of 200 mg/L was prepared. A mass of 100 mg TiO_2_/DBP was added to 50 mL of the silver nitrate solution with a specific concentration of Ag ions. Two different concentrations of Ag ions, 5% and 10% relative to the weight of the diatom biomass, were used for the synthesis of the AgNPs/TiO_2_/DBP composite. The impregnation process was carried out at room temperature for 12 h. 

During the interaction between the silver nitrate solution and pyrolyzed diatom biomass, following the principles of the inorganic chemistry of silver, it was assumed that the silver ions (Ag) in an alkaline environment would undergo oxidation to form silver oxides. The reaction can be represented as:

2AgNO_3(aq)_ + 2NaOH_(aq)_ → 2NaNO_3(aq)_ + Ag_2_O_(s)_↓ + H_2_O_(aq)_ (1)

The resulting silver oxide was then reduced using hydrogen peroxide as the reducing agent. The reducing agent was added in a molar ratio of 1:3 relative to AgNO_3_/H_2_O_2_. The mixture was stirred at 300 rpm for 15 min until complete reduction of the silver ions occurred:

Ag_2_O_(s)_↓ + H_2_O_2(aq)_ → 2Ag^0^_(s)_↓ + H_2_O_(aq)_ + O_2(g)_↑ (2)

The addition of the reducing agent led to a change in the pH of the solution from 9.02 to 6.88. Subsequently, the TiO_2_/AgNPs/DBP composite was washed five times with deionized water, centrifuged (Centrifuge 9000, MPW-251 rpm) and dried at 110 °C for 12 h.

### 2.3. Characterization Methods and Instrumentation

The scanning electron microscopy (SEM, LEO 1430 VP, Leo Electron Microscopy Ltd., Cambridge, Great Britain) coupled with energy-dispersive X-ray detection (EDX) (XFlash 4010, Bruker AXS, Berlin, Germany) and transmission electron microscopy (TEM, Tecnai F20 X-Twin, FEI Europe, Brno, Czech Republic) techniques were employed to determine the elemental composition, sizes, and distributions of the composite structures of the synthesized AgNPs/TiO_2_/DBP composites. TEM was utilized for imaging, while the STEM-EDS mode was employed for elemental analysis. The X-ray powder diffraction (XRD) method, using a Philips X’Pert Pro diffractometer (XRD, Malvern Panalytical Ltd., Malvern, UK) with Cu Kα radiation (γ = 0.1541 nm, 40 kV, 30 mA) was employed to determine the mineral composition of the obtained composites and investigate the formation of Ag and TiO_2_ nanoparticles. The XRD spectrum data were collected in the angular range of 5–85° 2θ with a step size of 0.01. For the investigation of the photoluminescent properties (PL) of the TiO_2_/AgNPs/DBP composites, a Hitachi F-2500 fluorescence spectrophotometer equipped with a xenon lamp was utilized. The PL spectra were recorded at room temperature using an excitation wavelength in the UV-VIS range from 200 to 750 nm. Measurements were conducted with a colloidal solution of the obtained material in water, with a concentration of 0.014 mg/mL, in a quartz cuvette with a diameter of 1.0 cm. The basic spectra were corrected accordingly. The obtained photoluminescent data were normalized using the Origin program. In addition, data after normalization were smoothed using the Adjacent Averaging method. 

### 2.4. Antimicrobial Potential Investigation of the Obtained Formulations

At this stage, three bacterial strains were selected, including two Gram-negative strains belonging to *Klebsiella pneumoniae* and *Escherichia coli*, as well as one Gram-positive strain, *Staphylococcus aureus*. The antimicrobial properties of the obtained composites were tested against a total of six strains, three of which were photogenic isolates obtained from the collection of the Centre for Modern Interdisciplinary Technologies (CMITC), and the other three strains were sourced from the American Type Culture Collection (ATCC) (Table 1).

The susceptibility of the selected bacterial strains was investigated using the minimum inhibitory concentration (MIC) assay, following the procedures outlined by the Clinical and Laboratory Standards Institute (CLSI). Two types of AgNPs/TiO_2_/pyrolyzed diatomaceous biomass composites (1.13% AgNPs/TiO_2_/DBP and 2.42% AgNPs/TiO_2_/DBP) were tested at various concentrations: 10 mg/mL, 5 mg/mL, 2.5 mg/mL, 1.25 mg/mL, 0.625 mg/mL, 0.312 mg/mL, and 0.156 mg/mL. The prepared concentrations were mixed in a 1:1 ratio with the cultured bacterial strain (1 × 10^6^ CFU/mL) and incubated at 37 °C. After 24 h, 12 μL of a resazurin-based in vitro toxicology assay kit (Sigma-Aldrich, St. Louis, MO, USA) was added to each well. According to the manufacturer’s recommendation, the MIC value was determined based on the colour change from blue to pink. Each experiment was performed in triplicate, and untreated cells and TiO_2_/pyrolyzed diatomaceous biomass composites served as control samples.

## 3. Results and Discussion

### 3.1. Energy-Dispersive X-ray Spectroscopy (EDS) Studies

**Figure 1:**
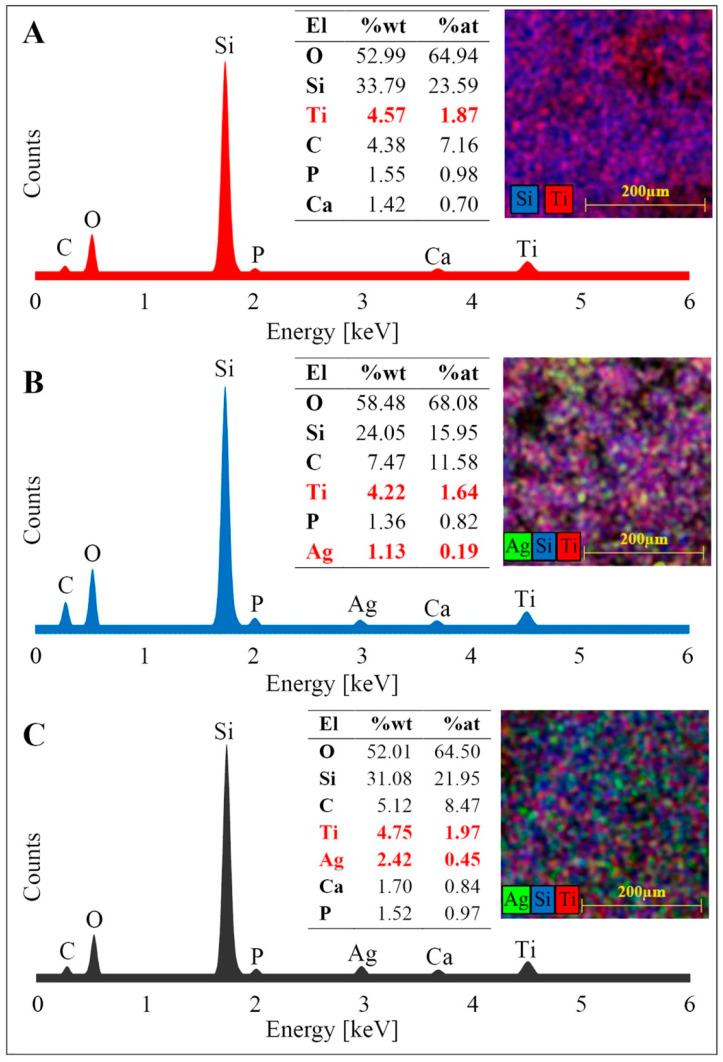
SEM-EDS spectra, elemental composition of composites and distribution maps of doped elements in composites: (**A**) diatomaceous biomass (DBP) with metabolically doped TiO_2_ (TiO_2_/DBP); (**B**,**C**) TiO_2_/DBP with chemically doped AgNPs (1.13% (**B**) and 2.42% (**C**), respectively).

The elemental composition of pyrolyzed diatomaceous biomass (DBP) with metabolically doped titanium dioxide (TiO_2_/DBP) and its silver AgNPs/TiO_2_/DBP composites was determined using patternless analysis SEM-EDS (Figure 1). This analysis revealed that the predominant components of the obtained materials were oxygen and silicon. The higher carbon content in the samples indicates the presence of the graphitized organic portion of the diatom cells after pyrolysis [39]. The presence of calcium and phosphorus may be attributed to these elements being associated with organic matter and remaining in their graphitized portion after pyrolysis. SEM-EDS analysis also showed a similar titanium content in all tested composites (average value of 4.51), suggesting a highly efficient process of TiO_2_ deposition on diatom cells.

The silver content in the AgNPs/TiO_2_/DBP composites varied due to the use of silver nitrate solutions with two different concentrations during the synthesis of metallic silver nanoparticles. Analysis of the weight percentages of silver in both composites (Figure 1B,C) indicates that the deposition process of AgNPs on diatomaceous biomass resulted in an efficiency of approx. 22.6% for the composite with a silver content of 1.13% AgNPs/TiO_2_/DBP and an efficiency of approx. 24.2% for the composite with a silver content of 2.42% AgNPs/TiO_2_/DBP. The silver-to-titanium ratio in the composites was 0.27 and 0.51, respectively. Krishnan et al. obtained an Ag:Ti ratio of 0.42 during the “green synthesis” of a bentonite-clay-supported silver (Ag)/titanium dioxide (TiO_2_) composite [40]. Mapping conducted for the obtained composites indicates a uniform distribution of titanium and silver in these materials.

### 3.2. Transmission Electron Microscopy Studies

**Figure 2:**
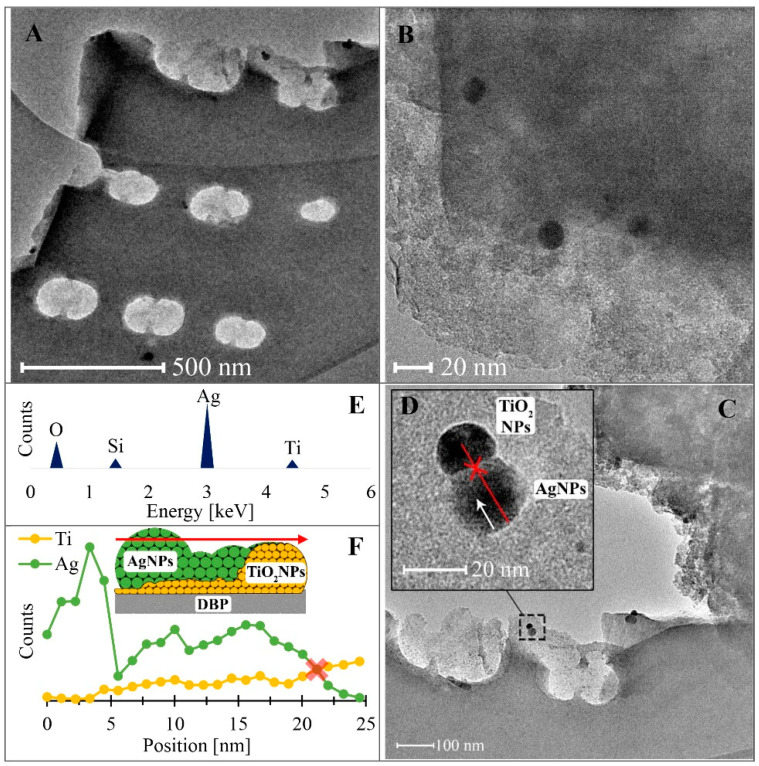
TEM images of the AgNPs/TiO_2_/DBP nanocomposite: distribution of AgNPs and TiO_2_ (**A**–**C**), single polycrystalline form of AgNPs/TiO_2_ (**D**), STEM-EDS AgNPs/TiO_2_ (**E**) spectrum, and STEM-EDS AgNPS/TiO_2_ line scan, along with the Volmer–Weber epitaxial growth model (**F**). DBP—diatomaceous biomass.

The results obtained from transmission electron microscopy (TEM) are presented in Figure 2. TEM micrographs (Figure 2A–D) show the distribution, morphology, shape, and dimensions of silver and titanium nanoparticles in the TiO_2_/DBP sample following silver adsorption from a solution with an initial silver concentration of 10 mg/L. It is evident that AgNPs and TiO_2_ nanoparticles exhibit evenly dispersed quasi-spherical forms on the surface of the pyrolyzed biomass. Interestingly, metallic silver and titanium dioxide nanoparticles often form hybrid twin pairs and are less frequently observed as individual nanoparticles. The STEM-EDS analysis (Figure 2E) of the interconnected nanoparticles (Figure 2D) further confirms that the larger nanoparticles primarily consist of metallic silver, while the smaller ones are composed mainly of TiO_2_. The relatively low silicon content may be attributed to the pyrolyzed biomass matrix [41]. The size of the silver nanoparticles observed in the TEM images (Figure 2D) ranges around 20 nm. This finding aligns with the synthesis conditions of our AgNPs synthesis, as Gontijo et al. [42] demonstrated that silver nanoparticles at pH ≈ 7 are typically around 20 nm in size. Other researchers investigating silver nanocomposites using various reducing agents and montmorillonite as a solid medium also obtained silver nanoparticles ranging from 2 to 50 nm [32,43]. The TiO_2_ nanoparticles, on the other hand, had an approx. size of 10 nm (Figure 2D). Furthermore, the elemental profile derived from the linear STEM-EDS scan (Figure 2F), conducted along a pair of hybrid AgNPs and TiO_2_ nanoparticles, reveals the heterogeneity in their composition and structure. According to the data from this profile, the nucleation and formation of AgNPs occur on previously formed TiO_2_ nanoparticles, a phenomenon known as epitaxy or epitaxial growth [44,45].

The epitaxial growth method is widely employed in the semiconductor industry to produce integrated crystalline layers of different materials [45]. The existing literature reports instances of epitaxial growth, such as SrZrO_3_ nanoparticles on the SrTiO_3_ substrate [46], Ag nanoparticles on Si nanowires [47], NiO nanoparticles on strontium titanate oxide [48], and single-crystalline graphene on hexagonal boron nitride [49]. The formation mechanism of such polycrystalline nanoparticle structures in our case (AgNPs deposition on TiO_2_) can be elucidated based on the classic nucleation theory, particularly the Volmer–Weber model of epitaxial growth, which is one of three primary epitaxial growth models (Volmer–Weber, Frank–van der Merwe, and Stranski–Krastanov) [50,51]. According to the Volmer–Weber deposition mode, the epitaxial layer originates from 3D nuclei on the growth surface of the substrate when the adsorbate–adsorbate interactions are stronger than the adsorbate–substrate interactions. Consequently, islands are formed through local nucleation, resulting in a non-planar epitaxial layer. The reaction pathway is driven by minimization of surface free energy [47,52].

### 3.3. Powder X-ray Diffraction Analysis

**Figure 3:**
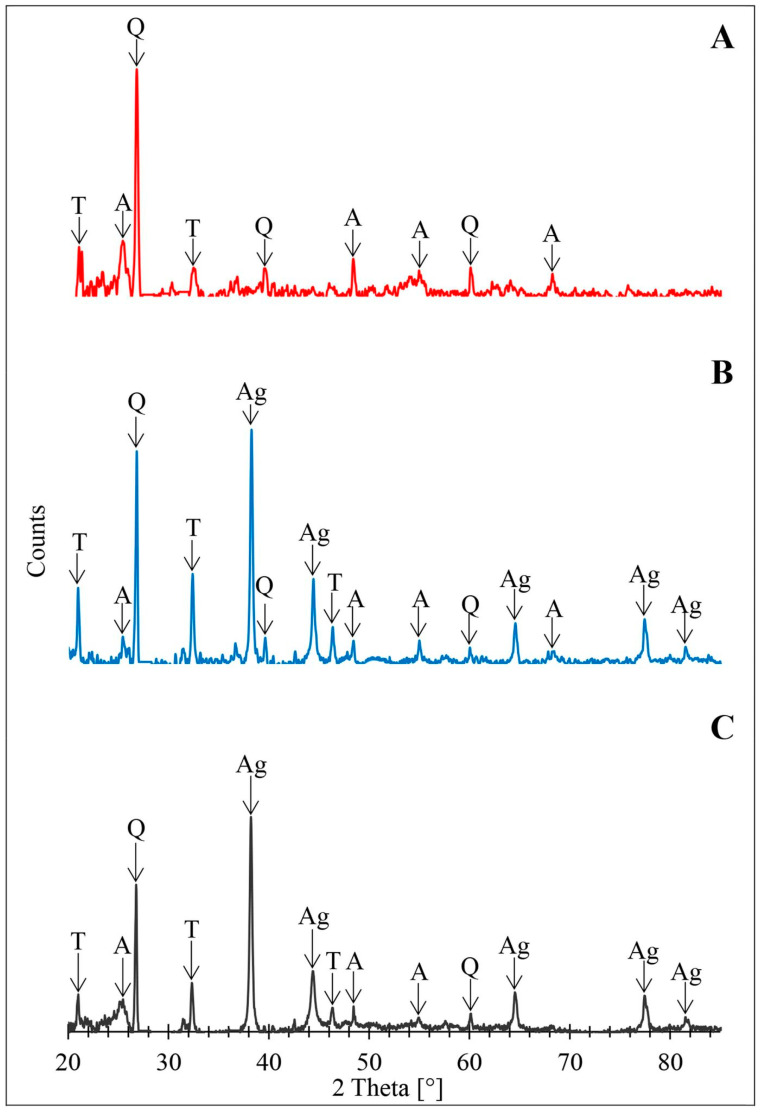
X-ray diffraction patterns of tested samples of pyrolyzed biomass (DBP): (**A**) with metabolically doped titanium (IV) oxide (TiO_2_/DBP), TiO_2_/DBP with doped silver: 1.13% (**B**) and 2.42% (**C**). T—tridymite, Q—quartz, A—anatase, Ag—metallic silver.

The X-ray powder diffraction patterns of pyrolised biomass immobilized with TiO_2_ (TiO_2_/DBP) and AgNPs/TiO_2_/DBP composites are depicted in Figure 3.

The X-ray spectra confirm the presence of TiO_2_ nanoparticles in the anatase form (Figure 3A–C), as well as reduced metallic silver nanoparticles (Figure 3B,C). The XRD spectrum obtained for TiO_2_/DBP (Figure 3A) exhibits characteristic peaks corresponding to the anatase phase of TiO_2_ at 2θ angles of 25.28° and 48.01° (RRUFF ID: R070582.9). Despite the pyrolysis temperature of 800 °C, which could suggest a transition from the amorphous titanium (IV) oxide to the crystalline rutile form, anatase proved to be the more stable form of TiO_2_ for nanoparticles [53]. This disparity arises from the influence of surface energy changes on phase transitions. In materials with a micrometric structure, the likelihood of a phase change is determined by the alteration of the selected thermodynamic function (free enthalpy, total energy change, entropy), which is defined by temperature, pressure, and number of moles, with changes in surface area being disregarded. However, in the case of nanomaterials, surface energy alterations exert a significant impact on phase transformations [54,55]. Moreover, the thermodynamic properties and associated phase transitions of nanomaterials are closely reliant on grain size, shape, and specific surface area. Anatase is the preferred form of titanium(IV) oxide for grain sizes smaller than 11 nm [55], such as those obtained in this study (Figure 2D). The spectrum displayed in Figure 3A also exhibits signals corresponding to crystalline forms of silica: quartz at 26.66°, 39.51°, and 60.02° (RRUFF ID: R040031) and tridymite at 19.28°, 21.64°, and 32.66° (RRUFF ID: R090042). It is now well-established that α-quartz attains stability at approx. 570 °C. Above this temperature, it undergoes a transformation to β-quartz, passing through at least one intermediate incommensurable phase within the temperature range of 573 to 574.1 °C [56]. At the pyrolysis temperature, there is a phase transition from β-quartz to β2-tridymite, which exhibits high stability up to 1470 °C, thereby accounting for the distinct peaks from both of these crystalline forms. Signals from anatase, quartz, and tridymite were also observed in the spectra presented in Figure 3B,C. Additionally, the aforementioned spectra display diffraction peaks at 38.17°, 44.35°, 64.48°, and 77.43° (RRUFF ID: R070416) which are consistent with the characteristic peaks of metallic silver.

### 3.4. Photoluminescence Properties

**Figure 4:**
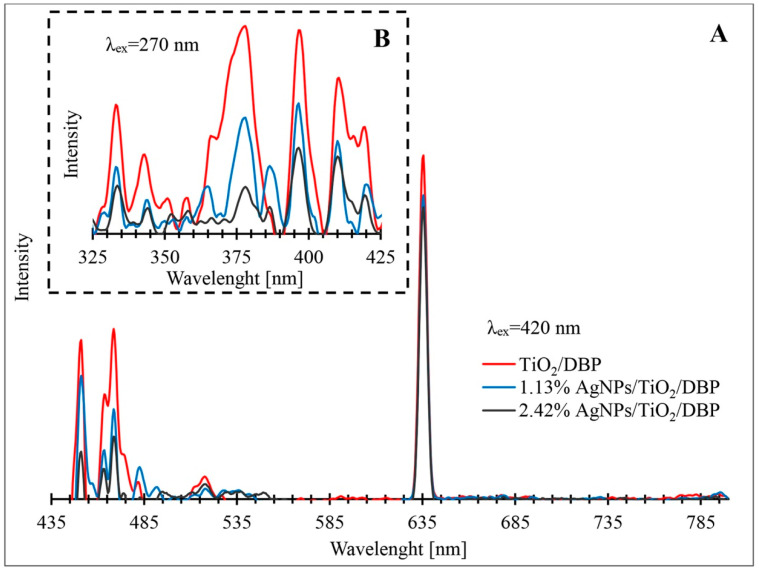
Photoluminescence emission spectra of pyrolyzed biomass with metabolically deposited TiO_2_ (TiO_2_/DBP) and its composites containing different concentrations of AgNPs: spectrum in the range from 435 nm to 800 nm, with an excitation wavelength of 420 nm (**A**), characteristic emission range of TiO_2_/DBP, at the excitation wavelength of 270 nm (**B**).

Figure 4 shows the photoluminescence of the pyrolyzed biomass with metabolically deposited TiO_2_ and its composites containing increasing concentrations of AgNPs. Analysing the obtained photoluminescence spectra, it should be noted that four main types of photoluminescence activity (PL) can be distinguished in all materials. The first type of PL is associated with excitation (270 nm) and emission in the ultraviolet region (325–425 nm). In this region, the most intense photoluminescence occurs at 333 nm, 343, 379, nm, 397 nm, 410 nm, 416 nm, and 419 nm (Figure 4B). The second type is associated with emission in the blue region of the visible spectrum (451–483 nm). Emission peaks at 451 nm and at 483 nm with 420 nm excitation are visible for all types of composites (Figure 4A). The third type of photoluminescent activity is characterized by emission in the green region (508–524 nm). In this region of the visible light spectrum, the most intense photoluminescence appears at a wavelength of 518 nm with an excitation of 420 nm. The final, fourth type of PL activity is associated with emissions in the red light region (630–640 nm). The most intense peak occurs at 635 nm with 420 nm excitation. The photoluminescence activity of the obtained composites in the ultraviolet region may be due to the fluorescence of the residual organic part in the pyrolyzed biomass [57,58]. The UV emission peaks ranging from 340 nm to 416 nm can be related to direct band transitions in TiO_2_, which affect the X edge of the Brillouin zone (BZ), namely the X_1b_ → X_1a_ and X_1b_ → Γ_3_ transitions, the intermediate transitions Γ_1b_ → X_2b_ and Γ_1b_ → X_1a_ [59,60], or may be due to self-trapped excitons or free excitons localized on octahedral TiO_6_ [61] resulting from the presence in the sample of the anatase form of TiO_2_ [62,63], confirmed by XRD studies (Figure 3).

Analysing the photoluminescence activity of the composites in the blue light region, two types of fluorescence emission peak were observed. These two fluorescence emission peaks correspond to the presence of AgNPs in the synthesized composites and are related to the relaxation of the electron motion of surface plasmons and the recombination of sp electrons with d-band holes [64]. In addition, the emission at 483 nm may be due to charge transfer from Ti^3+^ to the oxygen anion in the TiO_6_^8−^ complex associated with oxygen vacancies (V_o_) [65,66]. The origin of the intense emission in the green visible light ranges of the obtained composites may be the result of Si-O surface defects associated with nanoporous silica [67], as well as non-bridging oxygen hole centres (●O-Si≡), trapped excitons (-O-O-) and neutral oxygen vacancies (≡Si-Si≡) [68]. Garcia et al. [69] mentioned that the photoluminescence emissions observed in the visible spectrum may be due to medium-concentration recombination between the valence band and oxygen vacancies acting as donor levels. The nature of such emissions in the green range may be due to the photoluminescent activity of TiO_2_ nanoparticles in a narrow range of UV bands and the activation of biosilica oxygen vacancies, as well as the fact that TiO_2_ in the form of anatase also exhibits broad emission in the range of green light under UV excitation [60]. The emission in the red visible range at 635 nm can be attributed to oxygen vacancies associated with Ti^3+^ in the anatase form of titanium [59,60,61,65,66]. Oxygen vacancies in the TiO_2_ lattice are a type of internal defect that create intermediate energy states in the titanium dioxide bandgap. These oxygen vacancies act as photoinduced recombination centres for electron (e^−^) and hole (h+) pairs. Therefore, this emission occurred as a result of e^−^/h^+^ pair recombination through the oxygen gaps. In addition, a reduced emission intensity was observed for AgNPs/TiO_2_/DBP composites compared to TiO_2_/DBP without doping, which means that the doping of Ag ions effectively minimized the unfavourable e^−^/h^+^, recombination, resulting in more free charge carriers involved in photocatalytic reactions [70].

### 3.5. Antibacterial Activity

**Figure 5:**
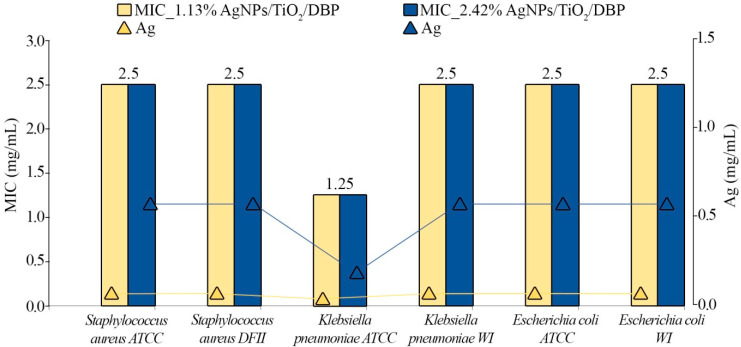
Antimicrobial activity potential of the obtained AgNPs/TiO_2_/DBP nanocomposites against selected bacterial strains. ATCC—American Type Culture Collection strains, DFI—diabetic foot infection isolate, WI—wound isolate, NI—no inhibitory effect.

Table 2 and Figure 5 present the MIC values of the two different nanocomposite formulations that incorporate different silver concentrations (1.13% AgNPs/TiO_2_/DBP and 2.42% AgNPs/TiO_2_/DBP) that have been investigated against selected bacterial strains.

The results indicate that all obtained nanocomposite formulations show antimicrobial potential against ATCC, DFI, and WI isolates exhibiting MIC values around 2.5 mg/mL against all tested strains, except for *Klebsiella pneumoniae* ATCC, where the MIC value was found to be one-fold lower (1.25 mg/mL). Compared to the TiO_2_/DBP nanocomposite without silver, the formulations that contained 1.13% and 2.42% of silver manifested antimicrobial potential against all investigated species.

It is worth noting that the results show independence of nanocomposite concentration, but dependence of silver content on antimicrobial potential. Therefore, it is suggested that the inhibitory effect was decided by the silver content (0.0283 mg and 0.0605 mg) incorporated in 2.5 mg nanocomposites. Moreover, although the MIC values of both investigated nanocomposites (1.13% AgNPs/TiO_2_/DBP and 2.42% AgNPs/TiO_2_/DBP) were found to be 2.5 mg/mL, the 0.0283 mg/mL of silver content was enough to inhibit the growth of the investigated ATCC, DFI, and WI strains. Only *Klebsiella pneumoniae* ATCC was more sensitive to the lower silver content (0.0142 mg/mL) compared to the rest of the investigated strains. It was not ruled out that the size of nanoparticles influenced the antimicrobial potential once the increase of the silver ion concentrations generated a higher size of nanoparticles.

Alternatively, the morphology, shape, and dimensions of silver and titanium nanoparticles in the TiO_2_/DBP sample can also decide the antimicrobial potential once larger nanoparticles consist mainly of metallic silver, and the smaller ones consist mainly of titanium dioxide TiO_2_. Furthermore, the hybrid nanoparticles are uniformly dispersed on the surface of pyrolised biomass and hybrid twin pairs are generated, as was recorded by microscopy.

A series of biochemical interactions explain the antimicrobial potential of silver formulations [70,71] However, the exact mechanism of action is still debated. The ability of the silver nanocomposite to damage the bacterial cell membrane is widely reported and it is considered the base mechanism. In turn, the impact of the secondary silver ions via a “Trojan-horse” mechanism has also been discussed [72]. In the context of nanocomposites with photocatalytic properties, the ROS species have been demonstrated to be the predominate mechanism that increased the antimicrobial activity through photoexcitation effects [73]. In the present research, the obtained AgNPs/TiO_2_/DBP formulations were found to show high photoluminescent activity. Therefore, it can be hypothesized that the antimicrobial effect is partially correlated with the photoluminescent activity. However, to prove such a theory, additional research should be designed and performed.

Many researchers demonstrated the potential application of silver formulations in pharmacology and cosmetology [74,75,76]. The incorporation of silver-based composites into ointments or hydrogels can avoid the delivery of free ions to the environment [75,77,78]. On the other hand, the synergistic effect of the silver nanoparticles against clinical isolates have been demonstrated [79]. S.A.H. Jalali et al. showed an increased antimicrobial effect after the addition of the TiO_2_ to the silver nanoparticles; the MIC value decreased from 0.0125 mg/mL and 0.025 mg/mL to 0.00625 (*E. coli*) and 0.012.5 mg/mL (*S. aureus*) [80]. The same phenomenon has been observed in the case of the present study, which demonstrated the increase of the antimicrobial effect of TiO_2_/DBP with the presence of silver. Furthermore, once the inhibitory effect was decided by the silver content and the effect was found to be one-fold higher compared to the literature data, we cannot exclude the fact that the diatomaceous biomass in which the silver content was incorporated could serve as a support for the controlled delivery system. Additionally, it is notable that three of the used bacterial strains in present research are clinical isolates and are considered to be pathogenic bacteria. Therefore, comparing our data to the literature results, we can firmly declare that the obtained formulations in the present study are of high interest. Moreover, the obtained silver formulations in the present study, based on TiO_2_/DBP, display higher antimicrobial potential against the same bacterial strains, compared to silver kaolinite nanocomposite where the MIC value was 10 mg/mL and silver content was 0.06 mg/mL [81]. Additionally, the present study shows almost the same antimicrobial activity reported by S.A.H. Jalali et al., even for the clinical strains [80]. This phenomenon can be explained by the synergistic effect generated by both silver and oxide titanium in the context of photoluminescent activity. Moreover, in the case of silver kaolinite nanocomposites [82], the antimicrobial potential appears to be dependent on the concentration, while in the case of silver TiO_2_/DBP, it does not.

Additionally, we would like to underline one more time that the obtained formulations were reported for the first time in this study. One aspect that makes them of high interest for both clinical and veterinary medicine is the fact that the support (diatomaceous biomass) used for the synthesis of the formulations is biodegradable and a good candidate for use in a controlled delivery system. Additionally, the smaller concentration of silver found to be effective for the clinical pathogens is another aspect that demonstrates the value of the obtained formulations.

## 4. Conclusions

A novel approach was developed for synthesizing hybrid AgNPs/TiO_2_/pyrolyzed diatomaceous biomass (AgNPs/TiO_2_/DBP) composites. The synthesis involved the metabolic doping of cultured diatom cells with titanium, pyrolysis of the doped biomass, and chemical doping of silver. SEM-EDX spectral analysis confirmed a uniform distribution of Ag and TiO_2_ nanoparticles in the resulting composites. X-ray diffraction confirmed the presence of TiO_2_ nanoparticles in the anatase form and AgNPs as metallic silver. TEM analysis revealed the epitaxial growth of AgNPs on the surface of TiO_2_NPs, forming Ag/TiO_2_ epitaxial hybrid nanoparticles. The introduction of doped silver led to a significant reduction in the photoluminescence intensity of AgNPs/TiO_2_/DBP composites, indicating a greater involvement of free-charge carriers in photocatalytic reactions.

Remarkably high antibacterial activity (minimum inhibitory concentration, MIC in the range of 0.01–0.06 mg/L) was observed for the synthesized nanocomposites against Gram-positive *Staphylococcus aureus* and Gram-negative *Klebsiella pneumoniae* and *Escherichia coli* strains, both laboratory-cultivated and clinical isolates. These findings provide valuable insights into the development of novel biocidal formulations to combat bacterial resistance and skin infections. Therefore, the nanocomposites obtained in this study hold promise as antimicrobial components in ointment and/or gel formulations. However, further investigations are necessary to assess the longevity and cytotoxicity of the nanocomposites in eukaryotic systems, such as cell lines and animal models.

## Figures and Tables

**Table 1:** The selected strains used in the present study for the antimicrobial potential investigation.

CMITC	ATCC
*Staphylococcus aureus* ATCC33591 THL (DFI), *Klebsiella pneumoniae* 9295_1 CHB (WI), *Escherichia coli* MB 11464 1 CHB (WI))	*Staphylococcus aureus* ATCC 700699, *Klebsiella pneumonia* ATCC 10031, *Escherichia coli* ATCC 10031

DFI—isolated from diabetic foot infection; WI—isolated from wound infection.

**Table 2:** The minimal inhibitory assay (MIC) AgNPs/TiO_2_/DBP. DFII—diabetic foot infection isolate; ATCC—American Type Culture Collection; WI—wound isolate.

Bacteria Strains		Minimal Inhibitory Concentration of AgNPs/TiO_2_/DBP [mg/mL]		
		TiO_2_/DBP	1.13% AgNPs/TiO_2_/DBP	2.42% AgNPs/TiO_2_/DBP
*Staphylococcus aureus*	ATCC	-	2.5 (0.0283 Ag mg/mL)	2.5 (0.0605 Ag mg/mL)
	DFII	-	2.5 (0.0283 Ag mg/mL)	2.5 (0.0605 Ag mg/mL)
*Klebsiella pneumoniae*	ATCC	-	1.25 (0.0142 Ag mg/mL)	1.25 (0.0303 Ag mg/mL)
	WI	-	2.5 (0.0283 Ag mg/mL)	2.5 (0.0605 Ag mg/mL)
*Escherichia coli*	ATCC	-	2.5 (0.0283 Ag mg/mL)	2.5 (0.0605 Ag mg/mL)
	WI	-	2.5 (0.0283 Ag mg/mL)	2.5 (0.0605 Ag mg/mL)

## Data Availability

Data will be made available on request.
